# An optimised direct lysis method for gene expression studies on low cell numbers

**DOI:** 10.1038/srep12859

**Published:** 2015-08-05

**Authors:** Anh Viet-Phuong Le, Dexing Huang, Tony Blick, Erik W. Thompson, Alexander Dobrovic

**Affiliations:** 1 University of Melbourne Department of Surgery, St Vincent’s Hospital, Melbourne, Victoria, Australia; 2 Translational Genomics & Epigenomics, Olivia Newton-John Cancer Research Institute, Melbourne, Victoria, Australia; 3 Institute of Health and Biomedical Innovation and School of Biomedical Sciences, Queensland University of Technology, Brisbane, Queensland, Australia; 4 St. Vincent’s Institute, Melbourne, Victoria, Australia; 5 Department of Pathology, University of Melbourne, Melbourne, Victoria, Australia; 6 School of Cancer Medicine, La Trobe University, Melbourne, Victoria, Australia

## Abstract

There is increasing interest in gene expression analysis of either single cells or limited numbers of cells. One such application is the analysis of harvested circulating tumour cells (CTCs), which are often present in very low numbers. A highly efficient protocol for RNA extraction, which involves a minimal number of steps to avoid RNA loss, is essential for low input cell numbers. We compared several lysis solutions that enable reverse transcription (RT) to be performed directly on the cell lysate, offering a simple rapid approach to minimise RNA loss for RT. The lysis solutions were assessed by reverse transcription quantitative polymerase chain reaction (RT-qPCR) in low cell numbers isolated from four breast cancer cell lines. We found that a lysis solution containing both the non-ionic detergent (IGEPAL CA-630, chemically equivalent to Nonidet P-40 or NP-40) and bovine serum albumin (BSA) gave the best RT-qPCR yield. This direct lysis to reverse transcription protocol outperformed a column-based extraction method using a commercial kit. This study demonstrates a simple, reliable, time- and cost-effective method that can be widely used in any situation where RNA needs to be prepared from low to very low cell numbers.

Circulating tumour cells (CTCs) are thought to play a critical role in cancer dissemination^1^,^2^. The detection and enumeration of CTCs offers prognostic value in many cancers including breast cancer^3^, prostate cancer^4^ and colorectal cancer^5^. Molecular characterisation of CTCs is an emerging field in cancer and holds great promise in assisting the current understanding of cancer metastasis^6^.

Reverse transcription quantitative polymerase chain reaction (RT-qPCR) has been regularly employed for the detection and characterisation of CTCs^7^,^8^,^9^,^10^. This technique allows the amplification from samples containing small amount of transcripts and can be multiplexed for the analysis of multiple gene targets simultaneously^10^. Since CTCs are rare in the bloodstream, optimising RNA yield from isolated CTCs prior to RT-qPCR is essential. As mRNA loss is likely to occur during column-based extraction, direct lysis methods in which reverse transcription can be performed directly on the cell lysate are an attractive alternative. Direct lysis methods also offer a simpler, cheaper and faster approach of preparing RNA to study gene expression profiles of CTCs, especially for single cells or small numbers of cells.

Eaton *et al.* was the first study reporting a direct lysis method for the analysis of isolated CTCs by RT-PCR^11^. It showed the compatibility of the plasma-membrane solubilising detergent Nonidet P-40 (octylphenoxy poly(ethyleneoxy)ethanol, sometimes called NP-40) with downstream RT-PCR. Since then, Nonidet P-40 has been utilised in other CTC studies across several cancer types, including colorectal cancer^12^, head and neck squamous cell carcinoma^13^ and breast cancer^14^,^15^. The original reagent has become unavailable and has been replaced with the chemically equivalent compound IGEPAL CA-630. A recent study by Svec *et al.* compared 17 different lysis solutions (including a IGEPAL CA-630 based lysis buffer) for a small number of astrocytes and found that a lysis solution containing bovine serum albumin (BSA) in water gave the best RT-qPCR performance^16^.

We had previously used a Nonidet P-40 based lysis solution that contained components of the subsequent reverse transcriptase reaction (RT mix A), including an RNase inhibitor and dithiothreitol (DTT) as RNA protective agents^11^,^12^,^14^,^15^. In the current study, we sought to test whether the BSA based lysis solution of Svec *et al.* was superior to IGEPAL CA-630 based lysis solutions for gene expression analysis by RT-qPCR from small numbers of breast cancer cells such as might be isolated after enrichment for CTCs, and whether a combination of the two (i.e., IGEPAL CA-630/BSA) might further improve the results. We compared lysis solutions (0.3% IGEPAL CA-630 alone, 0.3% IGEPAL CA-630 in RT mix A, 0.1% BSA alone, the combination of 0.3% IGEPAL CA-630 and 0.1% BSA, and water) for their capacity to release and protect mRNA from low cell numbers of different breast cancer cell lines and for their compatibility for downstream RT-qPCR. We demonstrated that IGEPAL CA-630/BSA lysis solution performed the best and was also superior to a commercially available column-based methodology for small numbers of cells.

## Results

### Comparison of lysis solutions for small numbers of MDA-MB-468 human breast cancer cells

We tested the ability of five different lysis solutions to lyse a small number of MDA-MB-468 breast cancer cells, and their compatibility with downstream RT-qPCR. The lysis solutions were 0.3% IGEPAL CA-630 in RT mix A, 0.3% IGEPAL CA-630 alone, 0.1% BSA alone, 0.3% IGEPAL CA-630 and 0.1% BSA combined, and water. “IGEPAL CA-630 in RT mix A” is a modification of our original lysis solution as per Eaton *et al.*^11^. It consists of SUPERase• In™ RNase inhibitor, DTT and pooled gene specific primers, rather than hexamers used in the original protocol. This was made with the rationale that Nonidet P-40 was established as an effective cell membrane lysis reagent and that SUPERase• In™ and DTT would help to protect the RNA from degradation after the cell membrane was lysed. Alternatively, IGEPAL CA-630 in water alone was added directly to cell pellets as used by Svec *et al.*^16^. Similarly, lysis solutions with BSA alone, IGEPAL CA-630/BSA, and water were used directly to lyse cell pellets and the RT mix was added 5 minutes after lysis on ice.

Each of the lysis solutions was applied to three cell pellets containing approximately one hundred cells, which had been collected by serial dilution and subsequent centrifugation (350 × *g* for 5 min). To minimise potential differences in cell inputs for each lysis solution, five samples of one hundred cells had been pelleted, from each of three replicate serial dilutions.

**Figure 1:**
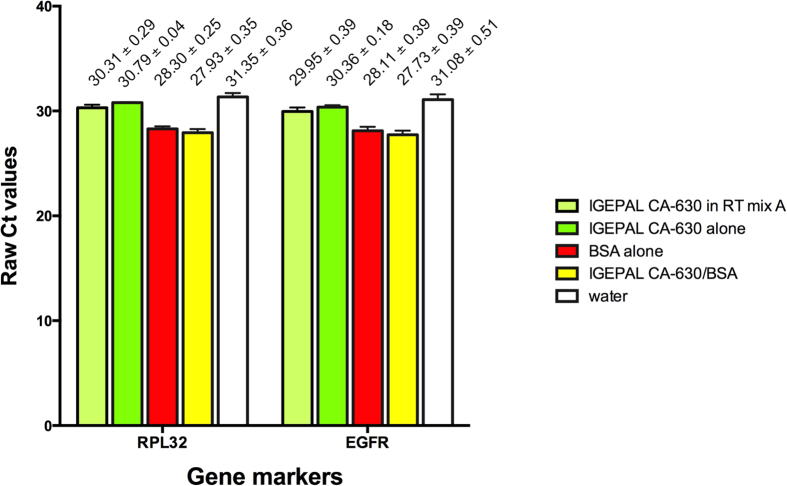
Comparison of five different lysis solutions for small number of MDA-MB-468 breast cancer cells. Three replicate samples of approximately one hundred cells were subjected to each different lysis solution, RT-qPCR was run and threshold cycle (Ct) values were determined for *RPL32* and *EGFR*. The earliest Ct values of IGEPAL CA-630/BSA indicate that it performed the best. Conversely, the latest Ct values for samples lysed in water indicate that it performed the worse. Lysis with BSA alone led to earlier Ct values than those obtained from lysis with IGEPAL CA-630 alone or IGEPAL CA-630 in RT mix. Error bars represent standard deviation (SD). Mean and SD values are shown on top of the corresponding bars.

Transcript levels of ribosomal protein L32 (*RPL32*) and epidermal growth factor receptor (*EGFR*), which are highly expressed in the MDA-MB-468 cell line, were measured by RT-qPCR. Highly expressed genes were chosen to avoid issues with stochastic gene expression, which can be a problem when working with very small numbers of cells. Sample preparation and the subsequent RT-qPCR were performed simultaneously to avoid batch-to-batch variation. The results shown in Fig. 1 are the average of the three replicates.

Consistent with the findings of Svec *et al.*^16^, the lysis solution with BSA alone gave earlier amplification than that with IGEPAL CA-630 alone (Fig. 1). The lysis solution that included components of the RT mix in addition to IGEPAL CA-630 performed slightly better than IGEPAL CA-630 alone (Fig. 1), but the difference was marginal. IGEPAL CA-630/BSA in the lysis mix gave the best RT-qPCR performance for both genes, although the improvement over BSA alone was marginal. This was more than 3 Cts better than water alone, an approximately 10-fold difference.

### Comparison of lysis solutions for small numbers of various human breast cancer cell lines

We expanded the comparison of the effectiveness of the lysis solutions to a further three breast cancer cell lines from several intrinsic subtypes^17^,^18^. BT549 and MDA-MB-231 are basal B breast cancer cell lines, MDA-MB-468 is a basal A breast cancer cell line and MCF7 is a luminal breast cancer cell line.

**Figure 2:**
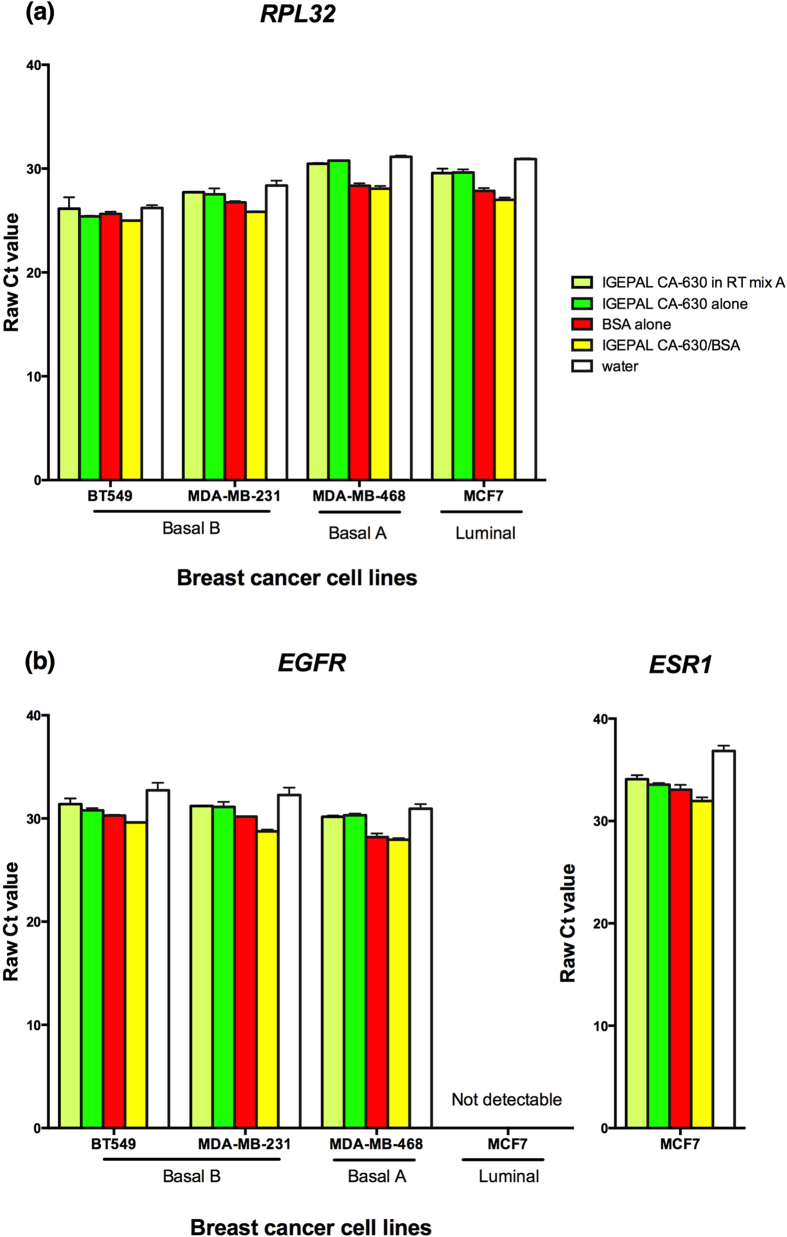
Comparison of different lysis solutions on small cell numbers of various breast cancer cell lines. For each cell line, replicates of approximately one hundred cells were subjected to each of the lysis solution. RT-qPCR was run and threshold cycle (Ct) values were determined for (**a**) *RPL32* and (**b**) *EGFR* or *ESR1*. In all cases, the lysis solution with IGEPAL CA-630/BSA gave the earliest Ct values, followed by that with BSA alone. Lysing with water alone gave the worst Ct values. Error bars are the standard error of the means (SEM) from two experimental replicates.

For each cell line, replicates of approximately one hundred cells were prepared by serial dilution, on which each of the five lysis solutions were tested in duplicate. RT-qPCR was performed for *RPL32* and *EGFR* for all cell lines. *EGFR* expression was not detected in MCF7 in our study, as expected, given its low to absent gene expression in luminal breast cancer cell lines^19^. Since MCF7 is an estrogen receptor positive cell line, we compared the five lysis solutions using RT-qPCR for estrogen receptor 1 (*ESR1*). The results, from two experimental replicates, across all transcripts and cell lines were consistent, in that water gave the highest raw Ct values (the worst RNA yield) for RT-qPCR, followed by lysis solutions containing IGEPAL CA-630 (alone and in RT mix). Once again, although the lysis solution containing BSA alone performed well, the IGEPAL CA-630/BSA lysis solution gave the lowest Ct values (Fig. 2). For both the *RPL32* and *EGFR* data, the difference in RNA yield between IGEPAL CA-630/BSA and each of IGEPAL CA-630 alone, IGEPAL CA-630 in RT mix, and water alone, as measured by RT-qPCR, was statistically significant in the post-hoc test following the repeated measures one-way ANOVA. In the same post-hoc test, the difference between IGEPAL CA-630/BSA and BSA alone was only significant in the EFGR data (p < 0.05).

### Comparison of direct lysis with column-based RNA extraction for small numbers of cells

**Figure 3:**
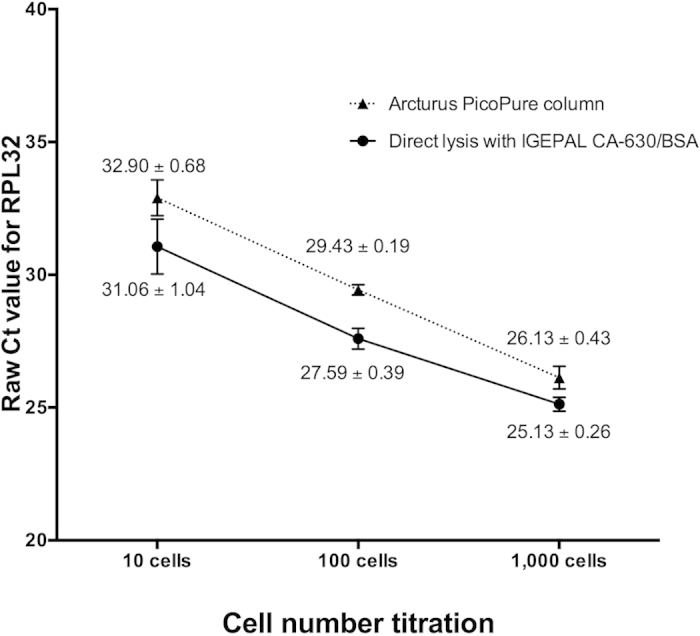
Comparison between direct lysis and a column-based RNA extraction for one hundred breast cancer cells. Direct lysis and column RNA extraction were each performed on two replicates of approximately one hundred MDA-MB-468 cells, followed by RT-qPCR *RPL32* and *EGFR.* The direct lysis method gave at least 1 Ct earlier than the column-based method. The experiment was performed twice. Error bars represent standard error of the means (SEM). Mean and SEM values are shown on top of the corresponding bars.

RNA extraction using the Arcturus PicoPure kit has been widely used to extract RNA from limited material obtained by laser capture microdissection^20^,^21^,^22^ and more recently from CTCs^23^,^24^. Since RNA extraction using a direct lysis method could minimise RNA loss and is more cost and time effective, we compared IGEPAL CA-620/BSA lysis solution, which gave the best results in the previous sections, against the Arcturus PicoPure kit. Initially, we applied these two methods on cell pellets containing approximately one hundred MDA-MB-468 cells. Raw Ct values obtained by RT-qPCR for *RPL32* and *EGFR* mRNA were used to compare the two methods. The experiment was performed twice, each with two replicates. We observed similar results for all repeats and replicates. Samples lysed directly with IGEPAL CA-630/BSA gave earlier Ct values, between one and two cycles, than those from the column extraction method, for both *RPL32* and *EGFR* (Fig. 3).

**Figure 4:**
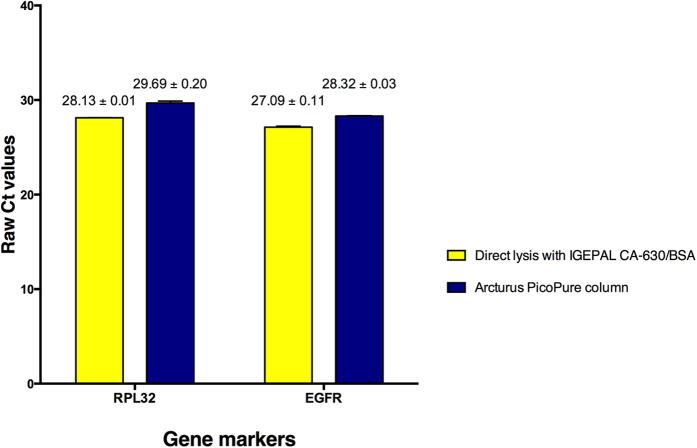
Comparison between direct lysis and column-based RNA extraction on a cell dilution series. Direct lysis and column-based extraction were compared across a range of cell numbers (10, 100, and 1,000 MDA-MB-468 cells), with two replicates at each cell number, for each method, and assessed by RT-qPCR. Direct lysis gave at least 1 Ct earlier than the column-based method. Error bars represent SD. Mean and SD values are shown on top of the corresponding bars.

We then compared the two methods across a range of cell numbers (10, 100, and 1,000 MDA-MB-468 cells), with two replicates at each cell number, for each method. Raw Ct values obtained by RT-qPCR for *RPL32* mRNA were used for the comparison. The relative effectiveness of the Arcturus PicoPure kit was consistent across the range of cell numbers tested, as reflected by the Ct values. At 10 and 100 cells in the dilution series, Ct values from directly lysed samples came up 1.8 cycles earlier than those from the Arcturus PicoPure column extraction method. At 1,000 cells, the difference in the mean Ct value was 1 Ct in favour of the lysis method (Fig. 4), indicating a possible decrease in efficiency of the lysis method at higher cell numbers. The difference in RNA yield, as measured by RT-qPCR, across the various cell numbers tested was statistically significant (p < 0.05).

## Discussion

Several methods that facilitate the isolation of RNA from very low numbers of cells for gene expression analysis have been published. Low numbers of cells require a procedure that minimises RNA loss during the RNA extraction step and thus gives the best RNA yield for RT-qPCR. Generally, direct lysis methods that avoid the use of columns and precipitation steps have been used to minimise the loss of RNA.

We compared direct lysis methods using low numbers of cells from several breast cancer cell lines by RT-qPCR. Our results showed that water alone gave the worst results, as would be expected. The lysis solutions containing IGEPAL CA-630 in water or in RT mix both performed markedly better, but could not be readily differentiated from each other. The lysis solution with BSA alone generally performed better than that with IGEPAL CA-630, consistent with the results by Svec *et al.* for astrocytes^16^, indicating that this is likely to be the case for multiple cell types. We further found that the combination of IGEPAL CA-630 and BSA gave improved RNA yields over either alone, as measured by RT-qPCR, across all the cell lines tested, from several intrinsic breast cancer subtypes.

Interestingly, our previous methodology using IGEPAL CA-630 and RNA-protective components of the RT mix did not perform better than the lysis solution using IGEPAL CA-630 alone (Fig. 1). It is however possible that the advantage of the protective reagents might be magnified under less optimal conditions than those used here, such as in long term storage of cell pellets, or samples with high RNase levels.

Since one of the most common approaches to extract RNA is column-based, we compared the IGEPAL CA-630/BSA lysis solution with the commercial extraction kit commonly used for small number of cells, the Arcturus PicoPure kit, and found an enhancement of at least 1 Ct. Since Ct values represent a negative log scale, at least one Ct earlier equates to at least double the RNA yield. Moreover, while the PicoPure extraction protocol alone takes a minimum of one hour, direct lysis method followed by RT reaction set up takes about ten minutes. In addition, IGEPAL CA-630 and BSA are low cost reagents. Thus the IGEPAL CA-630/BSA lysis method is faster, cheaper and more efficient than the column-based preparation.

The direct lysis method using the buffer containing both IGEPAL CA-630 and BSA is suitable for small number of cells, specifically one thousand cells or less. The line graph on Fig. 4 showed that the performance of the lysis method started to worsen at 1,000 cells, though it still gave better results than the Arcturus PicoPure column method. It has been shown that direct lysis with IGEPAL CA-630 works well up to a maximum of approximately one thousand cells^11^,^25^. Possible explanations for the decrease in the effectiveness of direct lysis on higher cell numbers include the higher concentration of RNases having a more marked impact, and other cell components reducing the RT efficiency. Thus, our method is not recommended for CTC samples enriched by a red blood cell (RBC) lysis method and density gradient centrifugation, due to a large amount of peripheral blood mononuclear cell (PBMC) contamination. On the other hand, CTC isolation by immunobeads and most current technologies using microfluidic CTC isolation, such as CTC-iChip^26^ or ClearCell Fx^27^, enable the isolation of single CTCs or pooled CTCs with the systemic removal of PBMCs and RBCs. Therefore, our direct lysis method is compatible and recommended for gene expression of CTCs isolated by such technologies.

In conclusion, we have found that a lysis solution composed of both of the mild non-ionic detergent IGEPAL CA-630 and BSA gives superior RNA recovery from low numbers of cells compared to previously reported solutions containing one or other of the components. In addition, direct lysis is a rapid, effective and economical alternative to a commercial column based extraction method when working with small cell numbers. This simple, reliable, time- and cost-effective method is suitable for gene expression studies of samples with low cell number, such as CTC studies, in which recovery can range from a single cell to several hundreds of cells.

## Methods

### Cell culture

The breast cancer cell lines used in this study (MDA-MB-231, MCF7, BT549, and MDA-MB-468) were from the American Type Culture Collection (ATCC, Rockville, MD, USA). MDA-MB-468 and MCF7 cells were cultured in Dulbecco’s Modified Eagle Medium with high glucose content (DMEM—Sigma-Aldrich, St. Louis, Missouri, USA) and supplemented with 10% Foetal Calf Serum (FCS—Sigma-Aldrich). MDA-MB-231 and BT549 were grown in Roswell Park Memorial Institute (RPMI)-1640 Medium (R8758, Sigma-Aldrich) supplemented with 10% FCS. All cells were grown in a humidified incubator (37 °C, 5% CO2).

### Cell number preparation

Cell samples containing approximately 10, 100 or 1,000 cells were prepared by serial dilution. The samples used to test conditions in parallel were prepared from one stock.

### RNA preparation

RNA from small number of cells was isolated by one of the two methodologies: (1) a column-based method with the Arcturus PicoPure RNA Isolation kit (Applied Biosystems — Foster City, California, USA) or (2) direct lysis methods as described below.

RNA extraction using the Arcturus PicoPure RNA Isolation kit was performed according to the manufacturer’s protocol. Briefly, 50 ul of extraction buffer was added to the cell pellet, followed by 30 min incubation at 42 °C. While the lysate was incubated, the column was wetted by adding 250 ul of condition buffer, followed by centrifugation at 16,000 × *g* for 1 min. 50 ul of 70% ethanol was then added to the lysate, mixed well and the whole mixture was transferred to the prepared columns. Columns were centrifuged for 2 min at 100 × *g*, followed by centrifuging at 16,000 × *g* for 30 sec to remove flow through. 100 ul of wash buffer 1 was added to the columns, which were then centrifuged for 1 min at 8,000 × *g*. After that, 100 ul of wash buffer 2 was added to the columns, which were then centrifuged for 1 min at 8,000 × *g*. Another 100 ul of wash buffer 2 was added to the columns. Columns were centrifuged at 16,000 × *g* for 2 min. Another spin at 16,000 × *g* for 1 min was performed to remove the wash residue in the columns. Purification columns were transferred to new Eppendorf tubes and 11 ul of elution buffer was carefully added onto the column membrane. Columns were centrifuged at 1,000 × *g* for 1 min, and subsequently at 16,000 × *g* for 1 min to elute RNA. 10 ul of RNA solution was obtained.

Direct lysis methods used lysis solutions of either 0.3% IGEPAL CA-630 (Sigma-Aldrich) alone, 0.3% IGEPAL CA-630 in RT mix A, 0.1% bovine serum albumin (BSA, Sigma-Aldrich), the combination of 0.3% IGEPAL CA-630 and 0.1% BSA, or water. 5 ul of each lysis solution (IGEPAL CA-630 alone, BSA alone, IGEPAL CA-630/BSA, and water) was added directly onto cell pellets, and the mixtures were incubated on ice for a maximum of 5 min. Another 5 ul of reverse transcription (RT) mix A (see below) were subsequently added to cell lysates to make a total of 10 ul. The mixture of cell lysate and RT mix were stored on ice until cDNA synthesis step. 10 ul of lysis solution with IGEPAL CA-630 in the RT mix A was added into the cell pellet, the mixture was on ice until cDNA synthesis step.

### cDNA synthesis (gene specific priming)

Gene-specific priming was used for all RT reactions. A standard reaction volume of 10 ul was used for the Arcturus PicoPure RNA Isolation kit and 20 ul if using direct lysis method for RNA preparation.

Briefly, for the 10 ul gene specific priming RT reaction volume (Arcturus kit), ‘RT Mix A’ comprised of 0.5 ul primer pool [8 uM] (primers from Geneworks—Adelaide, South Australia, Australia, or Integrated DNA Technologies — Coralville, Iowa, USA; 0.4 ul of dNTP [25 mM] (Bioline Reagents—London, UK), 0.5 ul SUPERase• In™ RNase inhibitor (Ambion—a Life Technologies subsidiary) and 0.6 ul of nuclease free water was prepared first. Half of the RNA extracted by the Arcturus kit (5 ul) was added to 2 ul volume of ‘Mix A’. 7 ul solution of Mix A and RNA was placed in a PCR block at 65 °C for 5 min. During this time, 3 ul of ‘RT Mix B’ including 2 ul of 5x Thermoscript cDNA synthesis buffer (Life Technologies), 0.5 ul of 0.1M DTT (Life Technologies) and 0.5 ul of Thermoscript RT enzyme (Life Technologies) was made. When the PCR block was cooled down to 55 °C, ‘RT Mix B’ was placed in the block separately for 2–3 min to achieve the hot-start. 3 ul of ‘RT Mix B’ was added to bring each reaction volume to 10 ul. The run was resumed and the 55 °C step was run for 1 hr, followed by 85 °C for 5 min and ending with 4 °C until collection. cDNA was stored long term at −20 °C or at 4 °C if it was used immediately.

In the 20 ul gene specific priming RT reaction volume (for direct lysis), volumes of reagents were adjusted such that the concentration of each component was same as the 10 ul gene specific priming RT reaction volume for Arcturus column-based method. The procedure for the RT reactions is as described above.

### RT-qPCR

All the quantitative PCR work was conducted on the Stratagene MX3000P (Stratagene, La Jolla, California, USA). Reactions were performed in 96-well plates; each well contained 20 ul quantitative PCR reaction. In brief, quantitative PCR setup initially involved the mixing of 2 x Premix and nuclease-free water, next the desired volume of each template was aliquoted into its respective tube and the premix/water mixture added to each template. Each 20 mL of 2 x Premix consisted of: 3.2 mL DMSO, 4 mL 10X PCR Gold Buffer, 12.08 mL nuclease-free H_2_O, 100 ul 1M Mg(CH3COO)2, 80 ul 100 mM dATP, 80 ul 100 mM dTTP , 80 ul 100 mM dCTP , 80 ul 100 mM dGTP, 1 ul SYBR® Green 1 and 200 ul AmpliTaq® Gold. For each gene, tubes were prepared and labeled for all the templates and non-template control and the appropriate volume of each primer pool was aliquoted into the labeled tubes. The premix/water/template was added into each respective tube. Once the premix/water/template/primer mixture was made, three aliquots of 20 ul were made from a given tube into each well of the 96-well plate as three technical replicates for PCR. In each 20 ul PCR reaction, there were 10 ul of 2 x premix, 1 ul of 5 uM primer pool, 0.5 ul cDNA template and 8.5 ul water. Thermal cycling conditions were 95 °C for 10 min, 95 °C for 30 sec followed by 60 °C for 1 min repeated for 40 cycles, and finally the dissociation step of 95 °C for 1 min followed by 60 °C for 30 sec and then 95 °C for 30 sec.

### Statistical Analysis

All statistical analyses were performed using GraphPad Prism 6. Data from the comparison of five lysis solutions across different cells lines was analysed by repeated measure one-way ANOVA, with each lysis solution compared to IGEPAL CA-630/BSA by the Holm-Sidak’s multiple comparison tests. Reported p values are multiplicity adjusted. Data from the comparison of the IGEPAL CA-630/BSA solution and the Arcturus PicoPure column across different cell numbers was analysed by paired t-test.

## Additional Information

**How to cite this article**: Viet-Phuong Le, A. *et al.* An optimised direct lysis method for gene expression studies on low cell numbers. *Sci. Rep.* **5**, 12859; doi: 10.1038/srep12859 (2015).

## References

[b1] Pantel K., Alix-Panabieres C. & Riethdorf S. Cancer micrometastases. Nature reviews. Clinical oncology 6, 339–351, 10.1038/nrclinonc.2009.44 (2009). 19399023

[b2] Lianidou E. S. & Markou A. Circulating tumor cells in breast cancer: detection systems, molecular characterization, and future challenges. Clinical chemistry 57, 1242–1255, 10.1373/clinchem.2011.165068 (2011). 21784769

[b3] Cristofanilli M. *et al.* Circulating tumor cells, disease progression, and survival in metastatic breast cancer. The New England journal of medicine 351, 781–791, 10.1056/NEJMoa040766 (2004). 15317891

[b4] de Bono J. S. *et al.* Circulating tumor cells predict survival benefit from treatment in metastatic castration-resistant prostate cancer. Clinical cancer research: an official journal of the American Association for Cancer Research 14, 6302–6309, 10.1158/1078-0432.ccr-08-0872 (2008). 18829513

[b5] Cohen S. J. *et al.* Prognostic significance of circulating tumor cells in patients with metastatic colorectal cancer. Annals of oncology: official journal of the European Society for Medical Oncology/ESMO 20, 1223–1229, 10.1093/annonc/mdn786 (2009). 19282466

[b6] Lianidou E. S., Markou A. & Strati A. Molecular characterization of circulating tumor cells in breast cancer: challenges and promises for individualized cancer treatment. Cancer metastasis reviews 31, 663–671, 10.1007/s10555-012-9366-8 (2012). 22692478

[b7] Fehm T. *et al.* Detection and characterization of circulating tumor cells in blood of primary breast cancer patients by RT-PCR and comparison to status of bone marrow disseminated cells. Breast cancer research : BCR 11, R59, 10.1186/bcr2349 (2009). 19664291 PMC2750121

[b8] Strati A. *et al.* Gene expression profile of circulating tumor cells in breast cancer by RT-qPCR. BMC cancer 11, 422, 10.1186/1471-2407-11-422 (2011). 21967632 PMC3224356

[b9] Usiakova Z. *et al.* Circulating tumor cells in patients with breast cancer: monitoring chemotherapy success. In vivo (Athens, Greece) 28, 605–614 (2014). 24982230

[b10] Sieuwerts A. M. *et al.* Molecular characterization of circulating tumor cells in large quantities of contaminating leukocytes by a multiplex real-time PCR. Breast cancer research and treatment 118, 455–468, 10.1007/s10549-008-0290-0 (2009). 19115104

[b11] Eaton M. C., Hardingham J. E., Kotasek D. & Dobrovic A. Immunobead RT-PCR: a sensitive method for detection of circulating tumor cells. BioTechniques 22, 100–105 (1997). 8994656 10.2144/97221st01

[b12] Hardingham J. E. *et al.* Molecular detection of blood-borne epithelial cells in colorectal cancer patients and in patients with benign bowel disease. International journal of cancer. Journal international du cancer 89, 8–13 (2000). 10719724

[b13] Winter S. C. *et al.* Long term survival following the detection of circulating tumour cells in head and neck squamous cell carcinoma. BMC cancer 9, 424, 10.1186/1471-2407-9-424 (2009). 19961621 PMC3087340

[b14] Raynor M., Stephenson S. A., Walsh D. C., Pittman K. B. & Dobrovic A. Optimisation of the RT-PCR detection of immunomagnetically enriched carcinoma cells. BMC cancer 2, 14 (2002). 12031094 10.1186/1471-2407-2-14 PMC115840

[b15] Raynor M. P. *et al.* Identification of circulating tumour cells in early stage breast cancer patients using multi marker immunobead RT-PCR. Journal of hematology & oncology 2, 24, 10.1186/1756-8722-2-24 (2009). 19500345 PMC2712470

[b16] Svec D. *et al.* Direct cell lysis for single-cell gene expression profiling. Frontiers in oncology 3, 274, 10.3389/fonc.2013.00274 (2013). 24224157 PMC3819639

[b17] Blick T. *et al.* Epithelial mesenchymal transition traits in human breast cancer cell lines parallel the CD44(hi/)CD24 (lo/-) stem cell phenotype in human breast cancer. Journal of mammary gland biology and neoplasia 15, 235–252, 10.1007/s10911-010-9175-z (2010). 20521089

[b18] Perou C. M. *et al.* Molecular portraits of human breast tumours. Nature 406, 747–752, 10.1038/35021093 (2000). 10963602

[b19] Hoadley K. *et al.* EGFR associated expression profiles vary with breast tumor subtype. BMC Genomics 8, 258 (2007). 17663798 10.1186/1471-2164-8-258 PMC2014778

[b20] Xu L. *et al.* Direct evidence that bevacizumab, an anti-VEGF antibody, up-regulates SDF1alpha, CXCR4, CXCL6, and neuropilin 1 in tumors from patients with rectal cancer. Cancer research 69, 7905–7910, 10.1158/0008-5472.CAN-09-2099 (2009). 19826039 PMC2859041

[b21] Bhati R. *et al.* Molecular characterization of human breast tumor vascular cells. The American journal of pathology 172, 1381–1390, 10.2353/ajpath.2008.070988 (2008). 18403594 PMC2329846

[b22] Mikulowska-Mennis A. *et al.* High-quality RNA from cells isolated by laser capture microdissection. BioTechniques 33, 176–179 (2002). 12139243 10.2144/02331md06

[b23] Nagrath S. *et al.* Isolation of rare circulating tumour cells in cancer patients by microchip technology. Nature 450, 1235–1239 (2007). 18097410 10.1038/nature06385 PMC3090667

[b24] Steinert G. *et al.* Immune escape and survival mechanisms in circulating tumor cells of colorectal cancer. Cancer research 74, 1694–1704, 10.1158/0008-5472.CAN-13-1885 (2014). 24599131

[b25] Fox B. C., Devonshire A. S., Baradez M.-O., Marshall D. & Foy C. A. Comparison of reverse transcription–quantitative polymerase chain reaction methods and platforms for single cell gene expression analysis. Analytical Biochemistry 427, 178–186 (2012). 22617801 10.1016/j.ab.2012.05.010

[b26] Karabacak N. M. *et al.* Microfluidic, marker-free isolation of circulating tumor cells from blood samples. Nat. Protocols 9, 694–710 (2014). 24577360 10.1038/nprot.2014.044 PMC4179254

[b27] Warkiani M. E. *et al.* Slanted spiral microfluidics for the ultra-fast, label-free isolation of circulating tumor cells. Lab on a chip 14, 128–137, 10.1039/c3lc50617g (2014). 23949794

