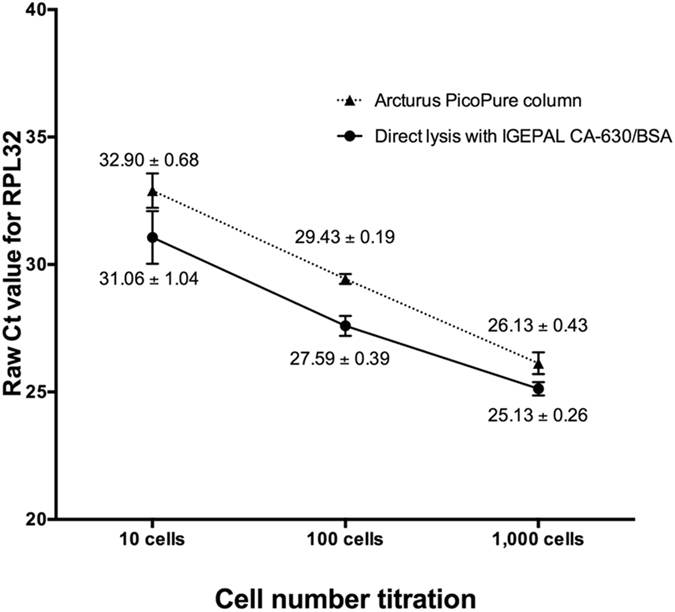# Erratum: An optimised direct lysis method for gene expression studies on low cell numbers

**DOI:** 10.1038/srep43075

**Published:** 2017-03-09

**Authors:** Anh Viet-Phuong Le, Dexing Huang, Tony Blick, Erik W. Thompson, Alexander Dobrovic

Scientific Reports 5: Article number: 1285910.1038/srep12859; published online: 08 05 2015; updated: 03 09 2017

This Article contains an error in the order of the Figures 3 and 4. Figures 3 and 4 were published as Figures 4 and 3 respectively. The correct Figures appear below as Figures 3 and 4. The Figure legends are correct.

## Figures and Tables

**Figure 3:**
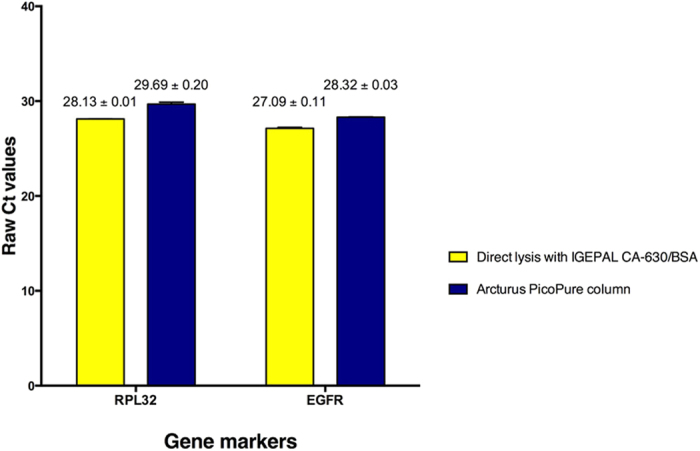


**Figure 4:**